# Analysis of New Colposcopy Techniques in the Diagnosis and Evolution of SIL/CIN: Comparison of Colposcopy with the DSI System (COLPO-DSI Study)

**DOI:** 10.3390/jpm13111605

**Published:** 2023-11-14

**Authors:** Virginia González González, María del Mar Ramírez Mena, Javier Calvo Torres, Miguel Ángel Herráiz Martínez, Irene Serrano García, Pluvio Coronado

**Affiliations:** 1 Ginemed Madrid Centro, 28036 Madrid, Spain; 2 Instituto de Salud de la Mujer, Hospital Clínico San Carlos, Facultad de Medicina, Universidad Complutense, 28040 Madrid, Spainpcoronadom@gmail.com (P.C.); 3 Research Methodological Support Unit, Hospital Clínico San Carlos, Instituto de Investigación Sanitaria del Hospital Clínico San Carlos de Madrid—IdISCC, 28040 Madrid, Spain

**Keywords:** colposcopy, cervical intraepithelial neoplasia, dynamic spectral imaging, dysis, hpv, human papillomavirus

## Abstract

Compared with conventional colposcopy, colposcopy assisted by DSI-map increases the detection of HSIL/CIN2+ and might help to identify the lesions more likely to regress. Introduction: Comparison of the performance of colposcopy assisted by dynamic spectral imaging (C-DSI) with that of conventional colposcopy (CC) in the diagnosis of cervical intraepithelial neoplasia (HSIL/CIN2 or CIN3). Materials and Methods: A total of 1655 women were referred for colposcopy between 2012 and 2020 and included in the study. Of that total, 973 were examined by the same colposcopist with C-DSI, and 682 with CC. Comparisons between CC and C-DSI were made by using the histological diagnosis performed with a punch biopsy or loop electrosurgical excision procedure (LEEP) as the gold standard. A follow-up study was conducted until 2021 to detect progression to HSIL/CIN2 at 6, 12 and 24 months after first examination. Results: C-DSI provided higher sensitivity for the diagnosis of HSIL/CIN2 or CIN 3 than CC (sensitivity of 76.8% and 86.6% vs. 54.2% and 72.2%, respectively). In negative or ASCUS/LSIL Pap smear results, C-DSI showed higher sensitivity than CC (sensitivity of 66.7% and 61.5% vs. 21.4% and 33.3%, respectively). In contrast, these differences were not observed in high-grade Pap smears. The sensitivity of C-DSI in cases with HPV16/18 infection was stronger than that of CC (73.53% vs. 56.67%). The sensitivity of C-DSI to detect the progression to HSIL/CIN2+ during follow-up was 30, 17.6 and 35.7% at 6, 12 and 24 months, respectively. Conclusions: The present study shows that C-DSI in women referred for colposcopy increases the HSIL/CIN 2–3 detection rate compared to conventional colposcopy. Nevertheless, C-DSI does not seem to be an important tool to predict the evolution of the lesions during follow-up.

## 1. Introduction

According to the International Agency for Research on Cancer (IARC) of the World Health Organization (WHO), cervical cancer was the fourth most common cancer among women in 2020 [1]. Cervical cytology has been the main cervical cancer screening test for most European and American societies. Nonetheless, the sensitivity of conventional cytology for detecting cervical intraepithelial neoplasia 2 (HSIL-CIN2+) does not exceed 80% under the best quality conditions [2]. The poor sensitivity and reproducibility of cervical cytology explains the recent focus on the role of HPV testing as an initial screening tool in the secondary prevention of cervical cancer [3]. Women with abnormal cervical screening results or suspicious symptoms are referred to conventional colposcopy (CC). The colposcopy is an essential tool for the diagnosis of preneoplastic lesions and a key instrument for secondary prevention of cervical cancer. Additionally, it represents a second-line technique in women with abnormal cytological results. It evaluates the presence, location, degree, extent, characteristics and type of precancerous or cancerous lesions. Furthermore, it helps to identify areas with atypical appearance in which biopsy would be useful and to determine the most appropriate treatment [4,5]. Several efforts have been made to improve the accuracy of colposcopy. Recent studies using the colposcopy-directed biopsy (CDB) show that almost 70% of the patients have high-grade intraepithelial lesions (HSIL). The outcomes obtained by LEEP conization show that half of the women are diagnosed with HSIL and one-third with the low-grade squamous intraepithelial lesion (LSIL) [6,7].

In order to improve the accuracy of colposcopic procedures, a dynamic spectral imaging system (DSI by DSI Medical Ltd., Livingston, UK) was developed. This device has been reported to be more sensitive than CC in detecting high-grade lesions and to provide a better selection of areas where taking biopsies might be useful. The Dynamic Spectral Imaging system (DySIS) performs a quantitative and cartographic measurement of the light scattering of cervical epithelium. When acetic acid is administered on atypical cervical tissue, the light-scattering properties of cervical epithelium change, switching its color into white (acetowhitening). This histological reaction is compatible with different lesions such as CIN-SIL, metaplasia, inflammation, epithelium repair or HPV infection. One of the main difficulties when cervical epithelium is evaluated with conventional colposcopy is to distinguish between these lesions, thus explaining the low sensitivity and specificity of the acetic acid cervical tests. Furthermore, these lesions often coexist, increasing the chances of biopsy sampling error. With the quantitative assessment of the dynamic optical changes, a more accurate location of cervical lesions would be possible. DySIS allows both CC and C-DSI evaluations to be performed with the mapping of cervical images. This system consists of an optical head, which is separated 25 cm from cervical tissue and provides uniform illumination with a white light. It also includes magnification optics, which are coupled to a digital camera. The camera is linked to a computer and a TFT monitor, which are essential for image display. After taking a reference image, 3% acetic acid is applied through a built-in applicator system, and then the images are taken every 5 s for 240 s from 23 × 20 mm cervical areas, including the transformation zone. For image acquisition, the system will use the timing and duration of the acetic acid on the cervix surface. As mentioned before, these images are then sent to a digital color camera. To avoid image darkening, linear polarizers should be placed in the imaging and illumination tracks. In turn, the camera is interconnected to a computer where the images are stored and processed with software. The system has a professional program for describing and archiving colpograms. The software automatically aligns the captured images with pixel precision to correct for patient movements during the scan. With the images, diffuse reflectance versus time integral is obtained using curve modeling to obtain the spatial distribution of the cervical lesions, displayed as a pseudo-color map overlaid onto the image of the cervix. Diffuse reflectance and time integral are obtained for every pixel image to mark atypical areas with colored circles that indicate possible biopsy sampling areas. Red, yellow and white mark the areas of high-grade disease, whereas green circles mark the biopsy areas selected by DySIS, and red circles the areas selected by the colposcopist [7].

The DSI mapping system (C-DSI) enhances the sensitivity for HSIL/CIN2+ detection, raising it to 86% compared to CC sensitivity [8]. The DSI color-coded mapping system could become an objective tool to assess and select the best sites for biopsy, thus reducing interobserver variability. It has also been described as an easier procedure than CC, especially among less experienced colposcopists [9]. Another benefit of this system is the computerization of the images and videos of the procedure, providing additional data for follow-up in conservative management and reducing overtreatment. However, some studies have reported potential misinterpretations of the reflection of the white light during the acetowhitening process or even an absence of an acetowhitening reaction during image recording when C-DSI is used [10]. In addition, failures have also been reported due to the application of acetic acid in concentrations other than 3%, movements during imaging despite the correction system, or the presence of mucus or blood in the cervix [7]. To the extent of our knowledge, previous studies using this technique were conducted by gynecologists with different qualification levels in colposcopy, leading to considerable heterogeneity and thus causing potential bias. A study developed in daily practice, together with a single, experienced colposcopist evaluation, would provide a realistic estimation of C-DSI performance. Colposcopy accuracy has a relevant role in the management of women with abnormal low-grade Pap-smear results, such as atypical squamous cells of undetermined significance (ASCUS) or low-grade squamous intraepithelial lesions (LSIL) [11]. In these cases, 10–15% of patients are finally diagnosed with HSIL/CIN2+ lesions after colposcopy and biopsy [11].

It would be challenging to evaluate the added value of the DSI mapping system in women with human papillomavirus (HPV) 16 or 18 infection. Different authors have stated that HPV 16 infection could be associated with more intense acetowhitening and more evident changes compared to HSIL/CIN2+ lesions associated with other high-risk HPV types (HR-HPV). Despite numerous factors that have been described to play an important role in colposcopy [4], the results of CC compared to C-DSI outcomes and their association with the evolution of the lesions in the long term have not yet been described. The aims of this study were (a) to analyze the detection rate of HSIL/CIN2+ by comparing the DSI system versus conventional colposcopy, (b) to determine if the DSI system increases the sensitivity of conventional colposcopy to detect high-grade lesions in women depending on their previous Pap-smear results or the presence of HPV, and (c) to evaluate its value in predicting the transformation of cervical lesions into HSIL/CIN2+.

## 2. Materials and Methods

An observational clinical study comparing conventional colposcopy with colposcopy used with the DSI system was performed. Women referred to our colposcopic unit at Hospital Clínico San Carlos, Madrid, Spain between January 2011 and June 2020 were recruited. We followed the Spanish Society of Cervical Pathology and Colposcopy (AEPCC) guidelines and indications to set the inclusion criteria [4,12], and those recommendations were still followed according to new updates to the guidelines [13]. These recommendations suggest that an immediate HSIL/CIN3+ risk of 5% or more should be used as the threshold to perform a colposcopy. Specifically, indications for colposcopy were HSIL, ASC-H, ACG, cervical adenocarcinoma in situ or invasive results in Pap smear; and high-risk HPV-positive test along with ASCUS or LSIL Pap smear. If patients had been under follow-up because of a previous high-grade lesion or if they had HPV 16/18, even with a negative Pap smear, colposcopy was also performed. Women with persistent HR-HPV infection were sent for colposcopy as well [4]. Women with high-risk HPV genotypes other than 16/18, no matter the genotype and even with a normal Pap smear, were only included if there was persistent infection (two positive test results separated by at least 1 year), as these patients had a lower immediate risk of HSIL/CIN3+ than women infected with 16/18 HR types [14]. Two cohorts were considered. The first cohort was composed of women who had been studied with C-DSI between January 2011 until June 2016. The second cohort was composed of women who underwent CC between July 2016 and July 2020. All included women were followed up until July 2021. All participants were assessed by a single expert colposcopist. The study was developed in accordance with STROBE recommendations and had the local ethics committee approval (C.I. 13/314-E). All patients signed the informed consent. Every recruited patient was managed and followed up according to the AEPCC guidelines [4]. Pregnant women and those with a history of pelvic radiotherapy or lower genital tract cancer were excluded. None of the patients had undergone colposcopy examination in the last 6 months. For the HPV-DNA testing, CLART^®^ HPV2 was used, which detects 35 different HPV genotypes. Genotypes 16, 18, 31, 33, 35, 39, 45, 51, 52, 56, 58, 59, 66 and 68 were considered HR-HPV. Negative detection and the low-risk HPV (LR-HPV) genotypes were classified as negative/LR-HPV.

**Figure 1:**
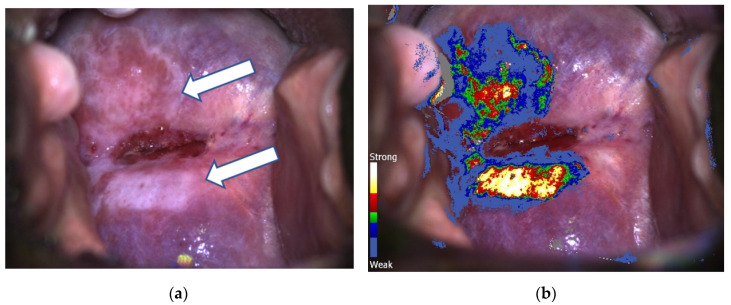
(**a**) Conventional colposcopy performed with acetic acid application. White arrows point to the areas with higher acetic acid absorption. (**b**) Colposcopy after obtaining the corresponding DySIS map of the cervix. Biopsies were taken from red, yellow and white areas. Histology confirmed a CIN2–3 lesion.

**Figure 2:**
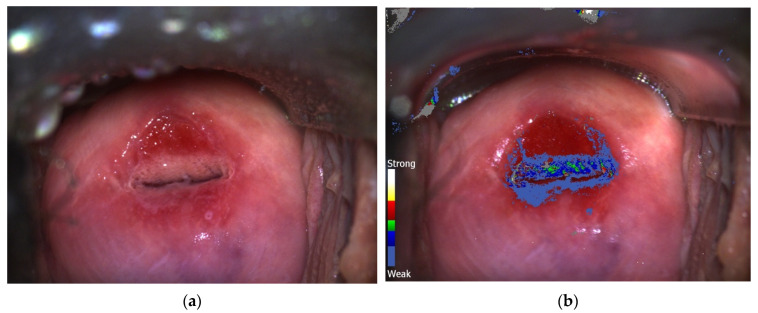
(**a**) Conventional colposcopy after acetowhitening. Notice acetowhitening process occurs in the anterior section of the cervix. (**b**) After performing DySIS-assisted colposcopy, green areas can be observed in the anterior section. Final biopsy result showed an LSIL/CIN 1 lesion.

Colposcopy and DSI examination were conducted as explained in previous articles [15,16,17]. Three-percent acetic acid was placed on cervical epithelium. The Schiller (iodine) test was performed if cervical lesions were not correctly identified with the acetic acid application. Or, if we could not find cervical lesions. During the 2–3 min the DSI mapping took to completion, the grade of cervical lesions and the location for biopsy were identified by CC. In turn, a color-coded DSI map was generated and interpreted. The blue colors marked normal tissue, whereas green indicated low-grade lesions (LG), and red-yellow-white colors suggested high-grade lesions (HG) [15]. Grey areas were interpreted as the presence of a large amount of fresh mucus in the cervical canal. For every case, a high-quality digital image of the CC was obtained by C-DSI technology, with its corresponding color-coded map (Figure 1a,b). In cases of discrepancy between CC and DSI interpretation, the final C-DSI decision was based on the highest grade obtained in one of the procedures (Figure 2a,b).

A cervical biopsy was taken with punch forceps if a cervical lesion was detected (either by CC or DSI), and an endocervical study was performed if the transformation zone was not entirely visible. If the C-DSI exam was normal, no random samples were taken, except when the Pap smear was positive for H-SIL, ASC-H or AGC. The loop electrosurgical excision procedure (LEEP) was indicated in patients with HSIL/CIN2+, in all cases of persistent LSIL-CIN1 for more than 2 years, or if there was discordance between the Pap-smear and colposcopy results (HSIL in cytology and LSIL/CIN1 or metaplasia in punch biopsy). If small and completely visible HSIL/CIN2 lesions were observed in women younger than 25 years of age, follow-up was considered in agreement with the patient instead of LEEP. When the punch biopsy and the LEEP specimen histology results were different, the higher grade was considered as the final pathology result for the patient. Pathologists examining biopsies were blinded regarding whether the biopsy was taken by using CC or C-DSI.

To analyze the progression of the cervical lesions, women who underwent LEEP or had an initial HSIL/CIN2+ diagnosis were excluded. Progression was considered when HSIL/CIN2+ was diagnosed during the follow-up at 6, 12 or 24 months.

## 3. Statistical Analysis

Qualitative variables were displayed with their frequencies and percentages, and continuous variables were calculated as mean and standard deviation. Sensitivity, specificity, predictive values and likelihood ratios with their 95% confidence intervals (95% CI) were calculated. A comparison of proportions was performed for independent samples to evaluate sensitivity and specificity significance. The association between the diagnostic tests in the detection of the final histologic outcome was calculated with Chi2 test. Every statistical test was two-sided. Statistical significance was defined as *p* < 0.05. Computations were developed using IBM SPSS Statistics version 25 (Chicago, IL, USA) and Epidat 3.1 (Galicia, Spain). The study protocol was accepted by the Clinical Research Ethics Committee of the Hospital Clínico San Carlos. (Reference number: 13/314-E; date of approval: 4 September 2013). All participants were asked to sign the informed consent.

## 4. Results

**Figure 3:**
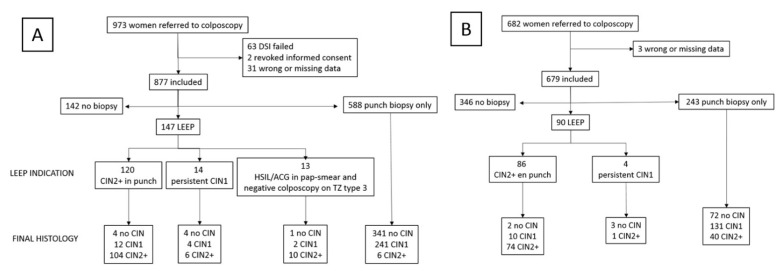
Flow chart of patients included and excluded. (**A**) Patients of C-DSI group. (**B**) Patients of CC group.

A total of 1655 women were recruited for the analysis: 973 in the C-DSI group and 682 in the CC group. For the follow-up study, 1487 patients were included, of whom 973 were studied initially with C-DSI, and 514 with CC. The median follow-up time for the C-DSI group was 43 months (ICQ 19–64), and 14 months (ICQ 7–25) for the CC group. The follow-up time was different because the two cohorts were recruited at different times. Figure 3 depicts a flow chart of included and excluded patients. In 63 patients, C-DSI failed due to intrinsic device failures caused by excessive movements during image taking or because of a software failure during the procedure that did not allow obtaining the color-map with precision. Patients without a biopsy record were considered as low-grade when the previous Pap smear was positive for ASCUS/LSIL and normal when the C-DSI and Pap smear were negative. A total of 147 patients in the C-DSI group and 90 in the CC group underwent LEEP. Table 1 represents the baseline characteristics of the sample. No significant differences were found in any of the variables studied. In the C-DSI group, low-grade abnormal changes were found in 477 women (49%), and high-grade abnormal changes in 130 women (13.4%). In the CC group, these findings were observed in 306 (45.1%) and 84 (12.4%) cases, respectively.

We performed 14 LEEPs in the C-DSI group due to persistent LSIL/CIN1 and four in the CC group (Figure 3), finding six cases (42%) of HSIL/CIN2 in the C-DSI group and one case in the CC group (25%) in the final histology.

Table 2A displays sensitivity, specificity, positive predictive value (PPV), negative predictive value (NPV) and positive (LHR+) and negative (LHR-) likelihood ratios for the detection of HSIL/CIN2+ and HSIL/CIN3+ lesions with both techniques. The sensitivity for detecting HSIL/CIN2+ and HSIL/CIN3+ was higher in the C-DSI group (76.8% and 86.8%, respectively) than in the CC group (54.2% and 72.2%, respectively). Table 2B shows these values for the detection of HSIL/CIN2+ depending on the previous Pap-smear result and HPV presence. Increased sensitivity for finding HSIL/CIN2+ with C-DSI was also identified in patients with normal or low-grade Pap smear (ASCUS/LSIL) (66.7% and 61.5% vs. 21.4% and 33.3%, respectively) and in patients with HPV 16/18 (73.5% vs. 56.67%) or HR-HPV (68.8% vs. 6.3%). Statistical significance was calculated, with significant differences in the sensitivity in the detection of HSIL/CIN2+ and HSIL/CIN3+ in colposcopy in combination with DySIS versus conventional; and in the detection of HSIL/CIN2+ based on the previous negative Pap smear, ASCUS/LSIL and HR-HPV (*p* < 0.001).

Non-significant values for sensitivity were obtained regarding the detection of HSIL/CIN2+ based on a previous HPV 16/18 test and ASC-H/HSIL/ACG/cancer smear (*p* = 0.008 and *p* = 0.895, respectively). Specificity significance was also calculated, with significant values regarding the detection of HSIL/CIN2+ and HSIL/CIN3+ in conventional colposcopy in combination with DySIS versus conventional colposcopy and the detection of HSIL/CIN2+ based on the previous Pap smear and ASCUS/LSIL Pap smear (*p* < 0.001). Detection of HSIL/CIN2+ based on a previous negative Pap smear and HR-HPV test was also significant (*p* < 0.05). In contrast, the detection of HSIL/CIN2+ based on a previous ASC-H/HSIL/ACG/cancer Pap smear and HPV 16/18 test did not reach significance (*p* = 0.491 and *p* = 0.098, respectively), see Table 2. Table 3 shows sensitivity, specificity, positive predictive value (PPV), negative predictive value (NPV) and positive (LHR+) and negative (LHR−) likelihood ratios for the detection of the lesion progression to HSIL/CIN2+ among the patients studied with C-DSI. The sensitivity ranged from 30% at 6 months to 17.6% at 12 months and 35.7% at 24 months. There were not enough cases of progression in the CC group to perform a statistical significance study.

## 5. Discussion

The HSIL/CIN2 detection rate is significantly higher when DSI mapping combined with CC is compared with CC alone. Previous studies have found a higher sensitivity of C-DSI to detect cervical lesions, particularly in the diagnosis of HSIL/CIN2+. C-DSI shows better performance in identifying biopsy sites compared to CC [8]. This is the second largest study [17] performed on DSI and the first focusing on the C-DSI utility in predicting cervical lesion progression.

The higher sensitivity of C-DSI obtained in the present study is consistent with the findings of previous studies [15,16,17,18,19,20] that documented an increased HSIL/CIN2+ detection rate of 20–30% and even up to 59% [8] while maintaining very high negative predictive value and thus helping to reduce overtreatment. The sensitivity of C-DSI obtained in our study was considerably higher than that observed by Booth et al. [21] (48.1%; 95% CI: 41.1–55.1) in the detection of HSIL/CIN2+. In that study, colposcopy procedures were performed by trained nurse colposcopists, residents and consultant physicians. However, in our study, C-DSI was carried out by an expert colposcopist, thus explaining the increase in the sensitivity due to a higher sensitivity in the associated CC. Harris et al. [22] reported an HSIL/CIN3+ detection rate of 2.5% when CC was used, increasing to 3.8% in the cohort of patients with ASCUS cytology if C-DSI-assisted colposcopy was performed. Those results mean a relative increase of 56.3% in the detection of HSIL/CIN3+. They observed a similar trend for the detection of HSIL/CIN2+, with a detection rate of 6.12% with CC and 9.38% with C-DSI, representing a relative increase of 53.3%. In our study, we also found a higher sensitivity with the C-DSI compared to CC, which is especially relevant in those with negative or ASCUS cytology. A substantial increase in the sensitivity to detect HSIL/CIN2+ was reported with C-DSI compared to CC in patients with normal or low-grade cytology. However, these differences were not seen in cases with HSIL in the Pap smear. This finding demonstrates the predictive power of C-DSI to detect the small high-grade lesions that had not been detected in cytology, whereas in high-grade Pap smears, in which the cervical lesions are more visible, no differences were found. In our study, endocervical curettage was performed when the transformation zone was not completely visible, according to the AEPCC guidelines [4]. Although endocervicoscopy can be potentially useful to diagnose and define endocervical lesions [23], it was not performed in our study. Its implementation is not included in the guidelines of the AEPCC, and it is not among the tests to be performed in the context of routine clinical practice. The sensitivity in detecting HSIL/CIN2+ lesions in patients with HPV 16/18 or HPV-HR infection was higher in C-DSI than in CC. Past studies have reported similar results with a higher detection rate in the case of HPV 16/18 (close to 100%) [17,20]. The authors suggested that HPV 16/18 infection could be related to the development of more acetowhite and long-lasting lesions than other HR-HPV genotypes. Zaal et al. [20] also described an increase in C-DSI sensitivity in detecting the progression to HSIL/CIN2+ in patients with HR-HPV (61%; 95% CI: 43–76 with CC versus 74%; 95% CI: 57–87 with C-DSI). The low sensitivity of C-DSI in detecting the progression to HSIL/CIN2+ during follow-up makes this technique not entirely suitable for these cases.

The strengths of this study are its large sample and the fact that exams were performed by a single senior expert colposcopist, with the same protocols and criteria for every patient. These increased the feasibility of the study and reduced bias. Additionally, the protocol of this study followed the daily routine clinical practice. There are some limitations related to the study. First, extrapolating the findings to other colposcopists might become a limitation. Another potential limitation is the moderate number of recruited biopsies and the fact that, in some patients, no punch biopsy was taken, because it was not considered clinically necessary, leading to possible HSIL/CIN2+ underdiagnosis. This weakness could bias the results, but not the relative differences between CC and C-DSI. The difference in the follow-up period between the cohorts could also be considered a limitation of the study. Because of this and since insufficient cases of progression were found in the CC group, a comparative analysis of both groups was not performed, and, therefore, a merely descriptive result of the presence of progression in the C-DSI group was presented. It would be interesting to extend the follow-up of the CC group to obtain a greater number of cases of progression, which will be considered in future research.

In conclusion, the use of DSI mapping in combination with CC exhibits a significantly higher HSIL/CIN2+ and HSIL/CIN3+ detection rate compared to CC alone. This study contributes to reinforcing the current scientific evidence related to this method and to confirming its feasibility in the prevention of cervical cancer.

## Figures and Tables

**Table 1:** Main characteristics of the cohorts.

	C-DSI Group N (%)/Mean (SD) N = 973	CC Group N (%)/Mean (SD) N = 682
**Age (years)**	35.16 (10.8)	35.7 (11.1%)
**Age at first sexual intercourse**	18.04 (3.0)	18.7 (9.6%)
**Anal sex**	138 (25.5%)	150 (22.1%)
**Current Smoker**	197 (20.2%)	154 (22.7%)
**Pap smear**		
Normal/ASCUS/LSIL	762 (83.4%)	566 (88.0%)
ASC-H/AGC/HSIL/Cancer	149 (16.4%)	77 (12.0%)
**CC/C-DSI result**		
Normal/Metaplasia	359 (36.9%)	284 (41.8%)
Low-grade abnormal changes	477 (49%)	306 (45.1%)
High-grade abnormal changes	130 (13.4%)	84 (12.4%)
Cancer	7 (0.7%)	5 (0.7%)
**HPV**		
Genotype 16/18	137 (14.1%)	113 (16.6%)
High-risk HPV no 16/18	199 (20.5%)	141 (20.8%)
Low-risk HPV	88 (9%)	73 (10.8%)
Negative	178 (18.3%)	70 (10.3%)
Undetermined	371 (38.1%)	282 (41.5%)
**Immunocompromised**		
HIV+	17 (1.8%)	1 (0.2%)
Other	25 (2.6%)	19 (2.9%)
**Patients who required LEEP**	162 (16.6%)	75 (10.9%)
Normal/Low grade	52 (5.3%)	35 (5.1%)
High grade	110 (11.3%)	40 (5.8%)

*p* > 0.05 in all variables. CC: Conventional colposcopy. C-DSI: CC assisted by dynamic spectral imaging system. SD: Standard deviation. ASCUS: Atypical squamous cells of undetermined significance. LSIL: Low-grade squamous intraepithelial lesions. ASC-H: Atypical squamous cells, cannot exclude HSIL. AGC: Atypical glandular cells. HSIL: High-grade squamous intraepithelial lesions. HPV: Human papillomavirus. HIV: human immunodeficiency virus.

**Table 2:** Sensitivity, specificity, predictive values and likelihood ratios in colposcopy in combination with DySIS versus conventional colposcopy. A: for the detection of HSIL/CIN2+ and HSIL/CIN3+; B: for the detection of HSIL/CIN2+ based on the previous Pap smear and HPV results. Data are given in percentages (95% confidence interval).

A. Detection of HSIL/CIN2+ and HSIL/CIN3+ in Colposcopy in Combination with DySIS versus Conventional						
Detection of HSIL/CIN2+						
	Sensitivity ***	Specificity ***	PPV	PNV	LHR+	LHR−
C-DSI (N = 856)	76.8 (69.4–84.2)	82.59 (79.8–85.4)	46.0 (39.3–52.5)	94.9 (93.1–96.7)	4.4 (3.7–5.3)	00.28 (0.2–0.4)
CC (N = 565)	54.2 (43.7– 64.7)	94.0 (91.7–96.3)	65.8 (54.7–76.9)	90.6 (87.9–93.4)	9.1 (6.0–13.7)	0.5 (0.4–0.6)
Detection of HSIL/CIN3+						
	Sensitivity ***	Specificity ***	PPV	PNV	LHR+	LHR−
C-DSI (N = 856)	86.8 (78.9–94.6)	79.4 (76.5–82.3)	31.2 (25.0–37.4)	98.2 (97.1–99.4)	4.2 (3.6–5.0)	0.2 (0.1–0.3)
CC (N = 547)	72.2 (59.4–85.1)	91.9 (89.4–94.4)	49.3 (37.7–61.0)	96.8 (95.1–98.5)	8.9 (6.3–12.5)	0.3 (0.2–0.5)

B. Detection of HSIL/CIN2+ based on the previous Pap-smear result and HPV presence.						
Negative Pap smear						
	Sensitivity ***	Specificity **	PPV	PNV	LHR+	LHR−
C-DSI (N = 74)	66.7 (0.0–100.0)	85.9 (77.1–94.7)	16.7 (0.0–41.9)	98.4 (94.5–100.0)	10.3 (1.0–105.1)	0.9 (0.7–1.1)
CC (N = 214)	21.4 (0.0–46.5)	97.9 (95.6–100.0)	42.9 (0.0–86.7)	94.4 (91.0- 97.9)	10.2 (2.5–41.3)	0.8 (0.6–11.1)
ASCUS/LSIL						
	Sensitivity **	Specificity **	PPV	PNV	LHR+	LHR−
C-DSI (N = 640)	61.5 (45.0–78.1)	82.8 (79.8–86.0)	18.9 (11.7–26.1)	97.1 (95.5–98.6)	3.6 (2.7–4.9)	0.4 (0.3–0.7)
CC (N = 274)	33.3 (17.3–49.4)	93.6 (90.3–97.0)	46.4 (26.2–67.0)	89.4 (85.4–93.5)	5.22 (2.7–10.1)	0.7 (0.6–0.9)
ASC-H/HSIL/ACG/Cancer						
	Sensitivity	Specificity	PPV	PNV	LHR+	LHR−
C-DSI (N = 139)	83.3 (75.4–91.3)	72.1 (57.5–86.7)	87.0 (79.5–94.4)	66.0 (51.4–80.6)	3.0 (1.8–4.9)	0.2 (0.1–0.4)
CC (N = 68)	83.7 (71.5–95.9)	66.7 (45.7–87.6)	81.8 (69.3–94.4)	69.6 (48.6–90.6)	2.5 (1.4–4.5)	0.2 (0.1–0.5)
HPV 16/18						
	Sensitivity	Specificity *	PPV	PNV	LHR+	LHR−
C-DSI (N = 117)	73.5 (57.2–89.8)	78.3 (68.8–87.8)	58.14 (42.2–74.1)	87.84 (79.7–96.0)	3.39 (2.2–5.4)	0.34 (0.2–0.6)
CC (N = 31)	56.67 (37.3–76.1)	94.12 (87.8–100.0)	81.0 (61.8–100.0)	83.1 (74.1–92.1)	9.6 (3.5–26.2)	0.5 (0.3–0.7)
HR-HPV						
	Sensitivity	Specificity	PPV	PNV	LHR+	LHR−
C-DSI (N = 176)	68.8 (42.9–94.6)	81.3 (74.9–87.6)	26.8 (12.1–41.6)	96.3 (92.7–99.9)	3.7 (2.3–5.8)	0.4 (0.2–0.8)
CC (N = 122)	6.3 (0.0–21.2)	93.4 (88.2–98.6)	12.5 (0.0–41.7)	86.8 (80.2–93.5)	1.0 (0.1–7.2)	1.0 (0.88–1.2)

PPV = Positive Predictive Value; NPV = Negative Predictive Value; LR (+) = Positive Likelihood Ratio; LR (−) = Negative Likelihood Ratio. High-risk HPV (HR-VPH) was considered the presence of any of the genotypes 31, 33, 35, 39, 45, 51, 52, 56, 58, 59, 66 and 68. * *p* < 0.05, ** *p* < 0.01, *** *p* < 0.001.

**Table 3:** Sensitivity, specificity, predictive values and likelihood ratios in DySIS to detect progression during follow-up.

Progression to HSIL/CIN2+						
	Sensitivity	Specificity	PPV	PNV	LHR+	LHR−
6 months	30 (0–63.4)	92.8 (83.8–100)	50 (1.7–98.3)	84.8 (73.3–96.3)	4.2 (0.9–17.8)	0.7 (0.5–1.1)
12 months	17.6 (0- 38.7)	74.3 (63.7–84.9)	13.6 (0–30.3)	79.7 (69.5–89.9)	0.7 (0.2–2.0)	1.11 (0.8–1.4)
24 months	35.7 (7.0–64.4)	75.5 (57.2–89.9)	35.7 (7.0–64.4)	75.5 (57.2–89.8)	1.4 (0.6–3.3)	0.9 (0.6–1.4)

PPV = Positive Predictive Value; NPV = Negative Predictive Value; LR (+) = Positive Likelihood Ratio; LR (−) = Negative Likelihood Ratio; HG = High Grade; LG = Low Grade.

## Data Availability

The data that support the findings of this study are available from the corresponding author, [VGG], upon reasonable request.
